# Biocultural Ethnoecology of *Nasua narica* (Procyonidae, Mammalia) in Northeastern Oaxaca, México

**DOI:** 10.3390/ani16162567

**Published:** 2026-08-18

**Authors:** Marco A. Vásquez-Dávila, Miguel Briones-Salas, Sergio García-Orozco, Rosa Elena Galindo-Aguilar, Gladys I. Manzanero-Medina

**Affiliations:** 1 Departamento de Ingenierías, Instituto Tecnológico del Valle de Oaxaca, Santa Cruz Xoxocotlán 71230, Mexico; 2 Instituto Politécnico Nacional, Centro Interdisciplinario de Investigación para el Desarrollo Integral Regional, Unidad Oaxaca, Santa Cruz Xoxocotlán 71230, Mexico; mbriones@ipn.mx; 3 Independent Researcher, Santa María Chimalapas 70360, Mexico; orozco_bioss@hotmail.es; 4 Centro Interdisciplinario de Investigación para el Desarrollo Integral Regional, Unidad Oaxaca, Secretaría de Ciencia, Humanidades, Tecnología e Innovación, Instituto Politécnico Nacional, Santa Cruz Xoxocotlán 71230, Mexico; rgalindoa@ipn.mx

**Keywords:** biocultural diversity, ethnozoology, indigenous hunting, traditional food systems, wild meat use

## Abstract

Oaxaca is one of the most biocultural diverse regions in Mexico. Although the white-nosed coati (*Nasua narica* L.; Procyonidae, Mammalia) is widely used by Indigenous and rural communities throughout the Neotropics, its role within subsistence hunting, traditional food systems, and local management practices in Oaxaca has remained undocumented. This study analyzes the biocultural significance of *N. narica* hunting and consumption among four Indigenous groups in northern Oaxaca. Data were collected through ethnographic techniques, i.e., semi-structured interviews with locals, ethnozoological field trips, and participatory workshops conducted with Zoque, Zapotec, Chinantec, and Mazatec communities. In addition, an extensive literature review was carried out to contextualize and compare the field data. *N. narica* hunting is predominantly diurnal and collective, frequently involving dogs (*Canis lupus familiaris*) and horses (*Equus ferus caballus*), and fulfills at least three functions, including food provisioning, crop protection, and limited wild meat trade. *N. narica* meat is incorporated into traditional diet and prepared in at least five culturally distinct culinary forms. The use of *N. narica* reflects complex ethnoecological knowledge and long-term human–fauna interactions embedded within Indigenous livelihoods. These findings underscore the relevance of a biocultural ethnoecological approach for understanding subsistence hunting, wild meat use, and rural livelihoods in Mesoamerica.

## Simple Summary

Across many rural regions of the world, wild animals remain an important source of food, income, and crop protection. Yet, the cultural meaning and everyday practices behind hunting are often poorly understood. This study examines the white-nosed coati (*Nasua narica*) and its role in the lives of Indigenous communities in northern Oaxaca, Mexico. Using interviews, field observations, and participatory workshops, we found that hunting is typically a collective activity involving dogs and horses, and it fulfills multiple roles, from providing food to protecting crops and occasionally generating income. The preparation of coati meat reflects a rich body of traditional culinary knowledge. More broadly, the use of this animal is embedded in systems of knowledge, values, and beliefs that shape how people relate to the natural world. Hunting, in this sense, supports the continuity of cultural traditions while guiding daily interactions with local ecosystems. Understanding these relationships is essential for developing biocultural conservation strategies that are both effective and respectful of local ways of life.

## 1. Introduction

Across rural landscapes worldwide, interactions between human societies and wild fauna are embedded within complex socioecological systems in which subsistence practices, cultural values, and ecological processes are closely intertwined [1]. From a biocultural ethnoecological perspective, hunting practices and wild meat consumption are expressions of long-term relationships between people and their environments, shaped by local ecological knowledge, cultural norms, and historical trajectories [2,3]. In many Indigenous and peasant societies, subsistence hunting contributes simultaneously to household nutrition, risk diversification, and the maintenance of cultural identity, while also reflecting adaptive responses to environmental variability and socio-economic change [4]. Consequently, the study of hunting and wild meat use offers a valuable analytical perspective into the co-production of biodiversity, livelihoods, and cultural practices in rural contexts.

Within this framework, previous research has emphasized the importance of documenting local hunting systems in situ, attending to their ecological foundations, social organization, and cultural rationales. In Mesoamerica, some studies [5,6,7] demonstrated how the use of wild meat in Indigenous communities is embedded in traditional knowledge systems, subsistence economies, and ethical norms regulating hunting practices. Their data highlight the need to situate hunting within broader ethnographic and ecological contexts, that is, within a biocultural ethnoecological framework [8]. However, despite the recognized relevance of such studies, important regional and taxonomic gaps persist. Medium-sized mammals such as the white-nosed coati (*N. narica*), which occupies an intermediate position between ecological functionality and cultural utility, remain underrepresented in ethnozoological research in southern Mexico. Addressing these gaps is especially pertinent in regions of high biocultural diversity, such as northern Oaxaca, where Indigenous peoples continue to rely on wild fauna as part of their rural livelihoods [9].

To situate this study biologically, it is necessary to briefly characterize the species involved. The family Procyonidae belongs to the order Carnivora and comprises 14 species distributed among six genera restricted to the Americas [10]. In Mexico, this family is represented by four genera and seven species, two of which are insular endemics [11].

**Figure 1:**
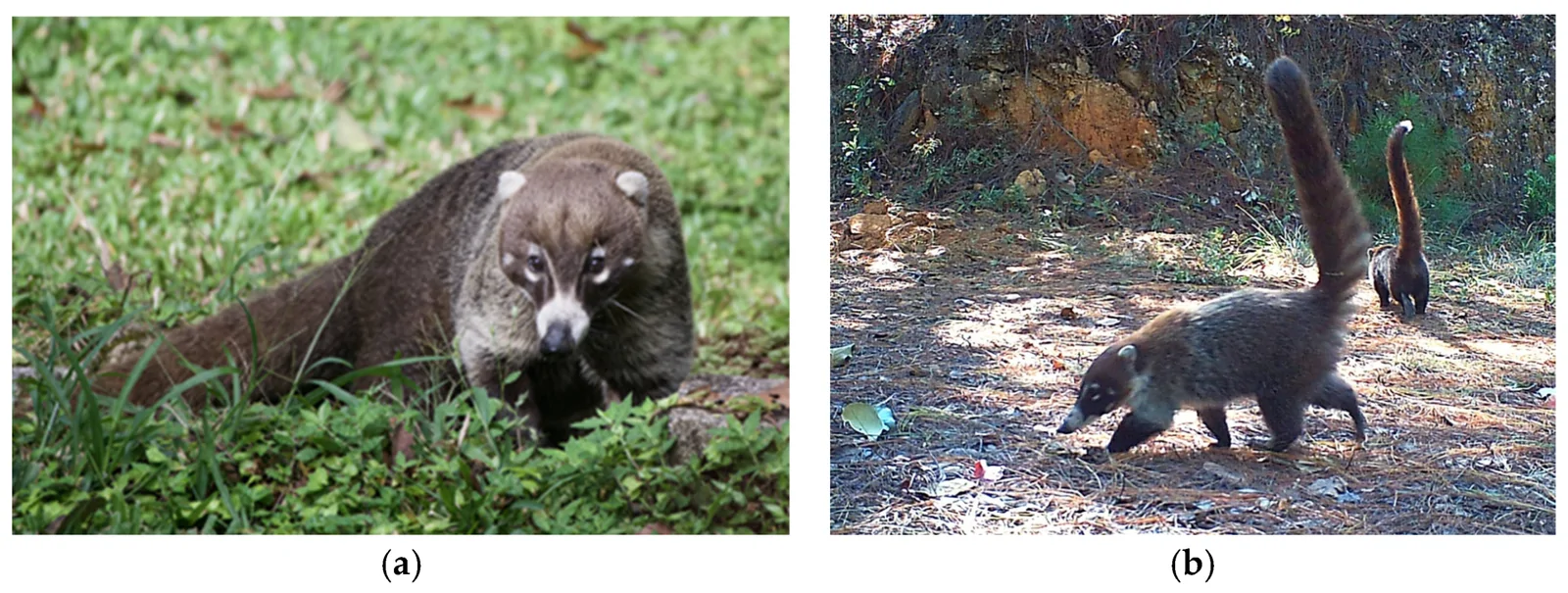
*N. narica* (Linnaeus 1766). (**a**) In Panama. (Photo: Dario Taraborelli, https://mexico.inaturalist.org/photos/174901321 accessed on 9 July 2026). (**b**) In Etla, Oaxaca (Photo: Eugenio Padilla Gómez).

The genus *Nasua* includes two species: *N. nasua* (Linnaeus 1766), distributed in South America, and *N. narica* (Linnaeus 1766; Figure 1), whose distribution extends from the southern United States (Arizona, New Mexico, and Texas) through Mexico and Central America to Panama and the western coast of Colombia [12]. In Mexico, *N. narica* is widely distributed, occurring in most regions except the Baja California Peninsula, the coastal plains of Sonora, and the driest areas of the Mexican Altiplano [13].

*N. narica* is a medium-sized mammal that has an omnivorous diet that includes fruits, insects, and small vertebrates [14]. Adult males are generally solitary [12], whereas females and juveniles form stable social bands [10]. Although capable climbers, *N. narica* is more commonly observed foraging on the ground [13]. Ecologically, *N. narica* plays an important role as a seed disperser [15] and has been reported as an accidental pollinator of pioneer tree species such as *Ochroma pyramidale* [14], which are important in processes of forest regeneration [16]. The species is also a prey item for large carnivores, including the cougar (*Puma concolor*) and the jaguar (*Panthera onca*) [17]. Additionally, a rare interspecific cleaning interaction has been documented in which *N. narica* removes and consumes ectoparasites from the tapir (*Tapirus bairdii*) [18].

*N. narica* exhibits a high degree of ecological plasticity and occupies a wide altitudinal range, from sea level up to 3500 m [10]. It inhabits both tropical and temperate forests, occasionally deserts and savannas, and has been recorded in human-modified environments such as agroecosystems [18]. *N. narica* is classified as a species of Least Concern [13]; however, local populations may be affected by habitat loss associated with deforestation and land-use change, as well as by hunting pressure in certain regions [13]. *Nasua* is by far the most commonly hunted genus of order Carnivora throughout Neotropics [19].

Oaxaca is recognized as the most biologically and culturally diverse state in Mexico, making it a particularly relevant setting for ethnobiological and ethnoecological research. In northern Oaxaca, Zoque, Chinantec, Zapotec, and Mazatec people have interacted with Neotropical ecosystems for centuries, developing complex bodies of knowledge, practices, and beliefs related to local fauna.

Despite the wide distribution of *N. narica* in Oaxaca and its recognized use by rural and Indigenous populations, no published studies have specifically examined its role in local food systems, hunting practices, or management strategies in the region. The objective of this study is to analyze the biocultural ethnoecology of *N. narica* through the documentation of traditional hunting practices, food uses, and management strategies in four Indigenous communities of northern Oaxaca, México, situating these practices within a broader regional context in Mexico and Central America.

## 2. Materials and Methods

This study employed a comparative ethnoecological approach combining site-based contextual description with qualitative ethnographic field methods. The methodological strategy was organized in two complementary components. First, the study sites are described to provide the ecological, geographic, and sociocultural context in which interactions between Indigenous communities and *N. narica* take place. Second, the qualitative research methods used to document hunting practices and food uses are outlined.

### 2.1. Field Sites

Santa María Chimalapa is located within the Isthmus of Tehuantepec region. The landscape includes the Sierra de Tres Picos, which rises to approximately 800 m above sea level, and the Sierra Atravesada, where the Picacho Prieto and Guayabitos hills are located [20]. The predominant climate is hot and humid, with rainfall occurring during both summer and winter and without a marked midsummer drought; winter precipitation accounts for approximately 10% more rainfall than in other regions [21]. On the Pacific slope of the Sierra Atravesada, climatic conditions vary according to altitude: above 500 m the climate corresponds to Aw2 in the Köppen classification, while lower elevations exhibit a drier Aw1 climate [21]. The main rivers draining into the Gulf of Mexico are the Corte and Uxpanapa, whereas the Chicapa and Ostuta rivers flow toward the Pacific Ocean. Vegetation types include tropical evergreen rainforest, montane cloud forest, and pine forest [22]. The municipality has 9578 inhabitants [23]. The Zoque language is currently spoken primarily by older adults, and the main economic activities are agriculture, livestock production, and forestry.

Santiago Camotlán is located on the Gulf slope of the Sierra Madre de Oaxaca and covers an area of 332.99 km^2^ [20]. Two climatic regimes are present: (a) a warm and humid climate with a mean annual temperature of 24 °C and average annual precipitation of approximately 4000 mm, and (b) a mild humid climate with a mean annual temperature of 20 °C and annual precipitation of about 3800 mm [21]. Vegetation types include cloud forest, tropical evergreen rainforest, oak forest, and pine forest [22]. The municipality comprises twelve settlements with a total population of 3346 inhabitants predominantly Zapotec speakers engaged in agriculture and extensive livestock production [23].

Ayotzintepec is on the Gulf slope of the Sierra Madre de Oaxaca, at an altitude of approximately 100 m above sea level. The municipality covers 169.69 km^2^ and, due to its proximity to Santiago Camotlán, exhibits similar climatic and vegetation conditions. The total population is 6857 inhabitants [23]. Chinantec is primarily spoken in the smaller localities surrounding the municipal center.

**Figure 2:**
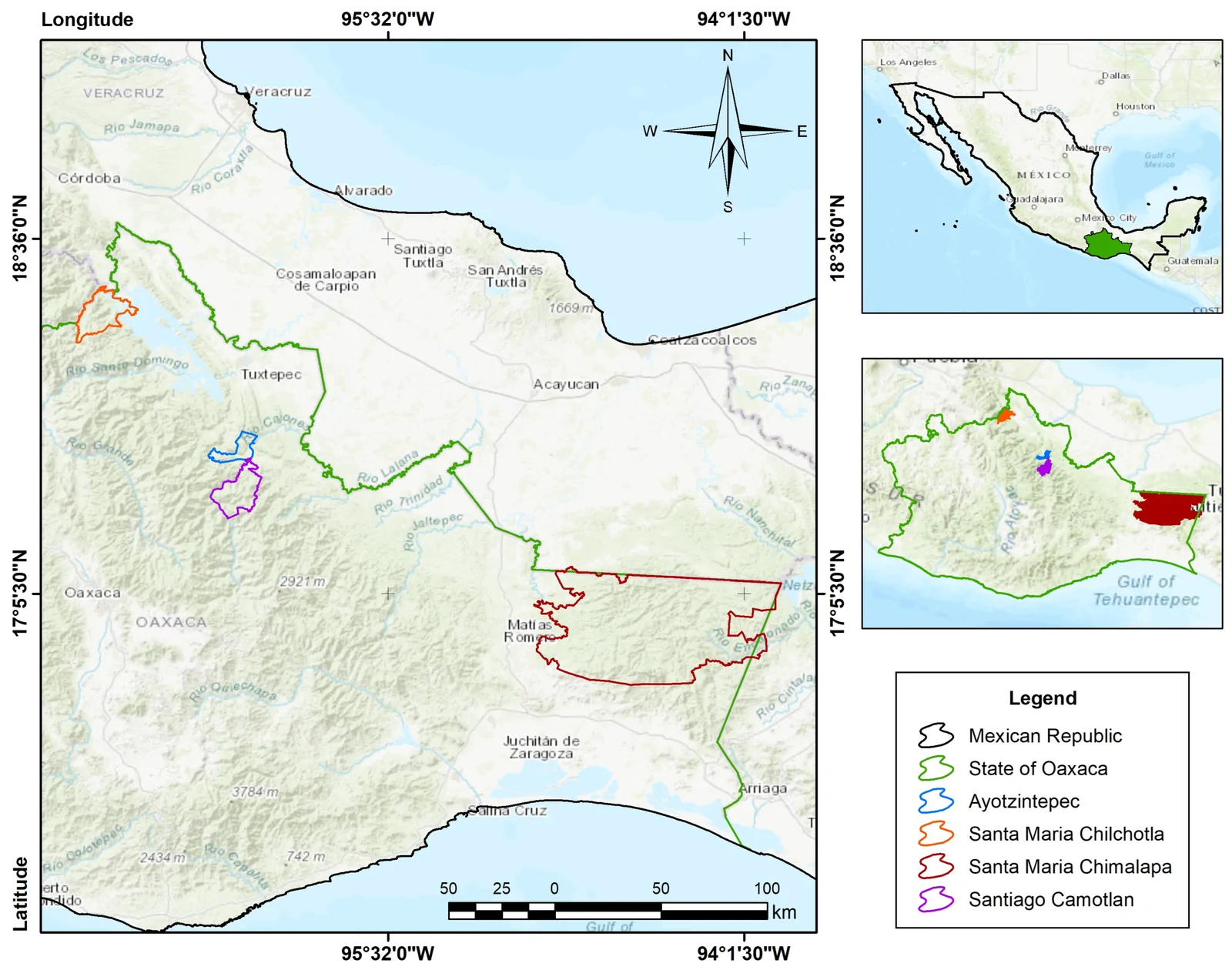
Field study sites in Northern Oaxaca, Mexico (Map elaborated by Rufino Sandoval-García).

Santa María Chilchotla is located in northern Oaxaca. Elevation ranges from sea level to 2100 m, and the municipality is commonly divided into high, middle, and low zones based on altitude and climate. Santa María Chilchotla has a total population of 21,469 inhabitants, predominantly Mazatec, distributed across 110 localities [23]. Figure 2 shows the four municipalities where fieldwork was conducted.

### 2.2. Fieldwork

Fieldwork was conducted in four Indigenous municipalities in northern Oaxaca: (1) Santa María Chimalapa, inhabited by Zoque people; (2) Santiago Camotlán, including the municipal seat and the locality of Arroyo Macho, with Zapotec speakers; (3) Ayotzintepec, including the municipal seat and the locality of Monte Mario, inhabited by Chinantec people; and (4) Santa María Chilchotla, including the localities of Barranca Seca, San Miguel Nuevo, Santa Eustolia, and La Luz, inhabited by Mazatec people.

Data were collected in the second 2025 semester through 48 semi-structured interviews with local knowledge holders (12 for each municipality). Interviews were open-ended and adapted to each participant to address five thematic domains: (1) hunting practices; (2) ecological knowledge; (3) food preparation; (4) meat sharing; (5) local management practices. In addition, 4 ethnozoological field trips and 4 participatory mini workshops complemented and triangulated the information gathered in the interviews.

Fieldwork conducted for this study adhered to ethical principles for research involving Indigenous Peoples and local communities. Prior informed consent was obtained through verbal agreements with community members, respecting local customs and norms. No specimens were collected, and data were generated through participant observation and informal dialogues with local actors. The study followed principles of recognition, reciprocity, and non-extractive engagement with traditional ecological knowledge [24]. The information obtained was analyzed comparatively and contrasted with published reports on the use of procyonids and other mammals by other Indigenous and rural groups in Mexico and Central America.

## 3. Results

This section presents the empirical evidence obtained through fieldwork in the four Indigenous regions studied. In accordance with the methodological approach outlined above, the results are organized into two main components. First, we describe hunting functions associated with *N. narica*, including subsistence hunting, crop protection (here conceptualized as vertebrate damage control), and limited commercialization. Second, we document the culinary uses of *N. narica* meat as a food resource, emphasizing local preparation techniques and cultural variability among ethnic groups.

### 3.1. Hunting Worldview, Knowledge and Practices

In Santa María Chimalapa, *N. narica* hunting is a diurnal and collective activity. On the evening prior to the hunt, hunters prepare their equipment, including firearms (16-gauge shotguns and 22-caliber rifles), sharpen machetes, and fill a bottle with sugarcane liquor. At dawn, dogs (*Canis lupus familiaris*) are fed and hunters share breakfast, an activity that local participants describe as reinforcing cohesion and mutual trust between men and the dog team. Meanwhile, one of the hunters brings horses (*Equus ferus caballus*) from the paddock, after which the group saddles up and begins the journey. During transit, dogs are tied to the horses’ tails with long ropes to prevent them from following other animal trails before reaching the hunting area.

Hunting areas are selected based on knowledge of the vegetation types frequented by *N. narica*, particularly the presence of fruiting trees, as well as on tracking signs. Upon arrival, the dogs are restrained while hunters perform a practice locally known as ***ensomar***, which involves rubbing a piece of cotton moistened with alcohol on the dogs’ noses “to clean them.” The dogs are then released into areas where *N. narica* tracks have been detected. Once the animal is located, hunters shoot it either in the forehead or in the thoracic region “below the armpit.” Large adult males are preferentially targeted because of their greater meat yield and firmer texture. Typically, one or two animals are hunted for consumption by a nuclear family; however, when a hunter from outside the family group participates, up to three individuals may be taken so that meat can be shared among households.

In Santiago Camotlán and Ayotzintepec, Zapotec and Chinantec participants reported *N. narica* hunting primarily when they damage maize (*Zea mays*) and banana (*Musa* spp.) crops. *N. narica* is locally recognized as a crop pest and, in some cases, as a predator of backyard poultry. This form of vertebrate damage control is understood as part of a broader set of traditional management strategies aimed at preventing or minimizing agricultural losses. Among the Mazatec communities of Santa María Chilchotla, *N. narica* is also considered a significant threat to maize fields. Local hunters observe and monitor *N. narica* groups to identify the moment when they enter cultivated plots, allowing hunters to intercept the animals and, in some cases, hunt up to five individuals during a single event.

In some contexts, *N. narica* hunting also fulfills an economic function. In Ayotzintepec and Santa María Chilchotla, *N. narica* meat was occasionally sold. In certain cases, groups of three to five hunters obtained multiple animals, generating income that exceeded the local minimum daily wage.

### 3.2. N. narica as Food Resource

In all four Indigenous regions studied, *N. narica* is consumed as food. In Santiago Camotlán, fresh meat is prepared in several ways, including cooking in its own juices, steaming, or preparing broth seasoned with chile (*Capsicum annuum* var. *annuum*). A dish locally referred to as ***barbacoa enchilada*** consists of meat cooked with chile, while sun-dried *N. narica* meat is used to prepare ***mole amarillo*** (yellow mole) a traditional Oaxacan dish. Yellow mole is a thick sauce made from cornmeal (*Zea mays*) and ***guajillo*** chile (*Capsicum annuum* var. *annuum*). It is flavored with ***pitiona*** (*Lippia alba*, Verbenaceae) and served with green beans (*Phaseolus vulgaris*), potatoes (*Solanum tuberosum*), and chayote squash (*Sechium edule*).

In Ayotzintepec, where *N. narica* is known in Chinantec as ***cha’kye***, preparation begins by singeing the animal’s hair over a traditional stove (***fogón*** in Spanish) and removing the remaining hair with a knife; the Mazatec communities follow a similar process. The meat is prepared in three main ways: (1) ***hoˉjnee***, a broth with ***guajillo*** chile and peppermint (*Mentha* × *piperita*); (2) ***naˉchuuy*’**, in which the meat is boiled and subsequently fried with tomato (*Solanum lycopersicum*) and ***guajillo*** chile; and (3) roasted, seasoned with crushed garlic (*Allium sativum*), chile, and salt.

Hunting strategies and culinary uses of *N. narica* across the four study localities are summarized in Table 1.

These findings, when considered collectively, suggest that the practices of hunting and consuming *N. narica* in northern Oaxaca are embedded within a more extensive biocultural system, wherein ecological knowledge, subsistence strategies, and cultural interpretations are intricately intertwined. The observed patterns—such as selective harvesting, the coordinated use of dogs and horses, and the diversification of culinary practices—reflects adaptive responses to local environmental conditions and forms of multispecies organization. At the same time, they reveal the presence of normative frameworks and shared values that regulate human–fauna interactions. In this sense, the use of *N. narica* extends beyond its material functions, constituting a culturally embedded practice that links livelihood strategies with cosmological understandings, behavioral ecology, and social norms, as widely documented in Mesoamerican hunting systems.

## 4. Discussion

Hunting in Mesoamerica is more than a subsistence activity; it constitutes a biocultural system through which ecological knowledge, cultural values, social institutions, and human–environment relationships are continuously reproduced [25,26]. Within this perspective, hunting cannot be understood solely as the extraction of wildlife but rather as a culturally regulated practice embedded in systems of knowledge, moral norms, and livelihood strategies that have developed through long-term interactions between Indigenous peoples and their environments [27,28]. The ethnographic evidence presented here shows that the use of *N. narica* in northeastern Oaxaca reflects this complexity. Hunting fulfills multiple and interconnected functions, including food provisioning, crop protection, limited economic exchange, and the maintenance of social relationships, while simultaneously reinforcing traditional ecological knowledge and cultural identity [29].

Our findings contribute to the growing body of biocultural ethnoecological research by documenting how a medium-sized carnivore is integrated into Indigenous hunting systems through a combination of worldview, ecological knowledge, specialized practices, cooperation with domesticated animals, food traditions, and locally embedded conservation principles. Rather than representing isolated behaviors, these dimensions constitute an integrated socioecological system that illustrates the adaptive relationships established between Indigenous communities and Neotropical ecosystems [5,6,7,8].

### 4.1. Worldview

The hunting of *N. narica* is embedded within a worldview in which forests are not merely sources of natural resources but living territories inhabited by beings endowed with agency and moral significance [30]. Within this cultural framework, hunting success depends not only on technical ability but also on maintaining respectful relationships with wildlife and the environments that sustain it. Animals are therefore perceived not simply as prey but as participants in a broader network of reciprocal interactions linking humans, non-human beings, and the landscape.

This worldview establishes implicit limits on extraction, discouraging excessive or wasteful hunting and reinforcing the idea that animals are temporarily “given” to humans [31]. The preference for hunting one or two individuals per expedition, increasing the number only when meat is shared among participating households, reflects this moral economy of restraint. In this sense, hunting constitutes an ethical encounter with the forest, regulated by culturally transmitted notions of reciprocity, moderation, and respect [32].

### 4.2. Ecological Knowledge

The hunting practices documented among Zoque hunters reveal a sophisticated body of traditional ecological knowledge regarding the behavior, habitat preferences, and seasonal movements of *N. narica*. Hunters identify productive hunting areas by recognizing vegetation types, fruiting trees, tracks, feces, and other environmental indicators that signal the presence of the species. This knowledge is cumulative, experience-based, and transmitted through continuous participation in hunting expeditions, illustrating the dynamic character of Indigenous ecological knowledge systems [32].

Ecological knowledge also guides harvesting decisions. Hunters preferentially target large adult males because of their greater meat yield and firmer texture, integrating empirical observations of the species with culinary preferences. Although motivated primarily by food quality, this selective harvesting may indirectly reduce pressure on reproductive females and illustrates how local ecological knowledge can influence resource use in ways that contribute to the persistence of wildlife populations [33,34]. Similar patterns have been documented among Indigenous hunting systems elsewhere in Mesoamerica, where detailed knowledge of animal behavior, habitat use, and life history underpins adaptive decision-making and reinforces long-term relationships between people and wildlife [5,6].

### 4.3. Crop Protection and Local Exchange

The findings demonstrate that the hunting of *N. narica* extends beyond food procurement and forms part of broader strategies through which Indigenous households manage interactions with wildlife in agricultural landscapes. In the Zapotec, Chinantec, and Mazatec communities included in this study, hunting frequently responds to crop damage caused by coatis, particularly in maize fields and banana plantations. Rather than representing a contradiction between wildlife use and conservation, these practices illustrate how local communities negotiate the coexistence of agricultural production and native fauna through adaptive management strategies developed over generations [5,6,7].

This dual role of *N. narica* as both a valued food resource and an agricultural pest reflects the complexity of human–wildlife relationships in Mesoamerican rural landscapes. Similar situations have been documented for other wild mammals, including peccaries, deer, armadillos, and pacas, whose management frequently combines subsistence hunting with the protection of crops and household resources [35,36,37]. Such patterns suggest that hunting decisions are rarely driven by a single motivation but instead emerge from the interaction of ecological conditions, subsistence requirements, and local perceptions of environmental change.

Occasional meat commercialization further illustrates this adaptive flexibility. Although market exchange represents only a minor component of *N. narica* use in the studied communities, the sale of surplus meat provides an additional source of household income without replacing the subsistence orientation of hunting. Rather than indicating a process of wildlife commodification, these small-scale exchanges reveal the capacity of Indigenous livelihoods to combine traditional practices with contemporary economic needs while maintaining local control over wildlife use [34].

### 4.4. Hunting Practices and Skills

The hunting practices documented in Santa María Chimalapa reveal a highly structured activity requiring technical expertise, ecological knowledge, and collective coordination. Hunting success depends on much more than marksmanship; it involves careful planning, the selection of appropriate hunting areas, the interpretation of environmental cues, and the coordinated participation of hunters, dogs, and horses throughout the expedition. These observations support previous ethnoecological studies showing that Indigenous hunting constitutes a complex body of practical knowledge acquired through long-term experience and intergenerational learning rather than a set of isolated technical skills [38].

The sequence of activities documented during fieldwork—including equipment preparation, the ritualized handling of hunting dogs, coordinated movement through the forest, and selective harvesting—demonstrates that hunting follows culturally standardized procedures that simultaneously maximize efficiency and reinforce collective participation. Such practices are transmitted through apprenticeship and repeated participation in hunting expeditions, allowing ecological knowledge to be continuously reproduced within local communities [32].

Viewed comparatively, these practices resemble those documented among other Indigenous peoples of Mexico and Central America, where hunting combines detailed environmental knowledge with socially organized labor and culturally regulated techniques [5,6,7]. Consequently, hunting should be understood not merely as a means of obtaining wild meat but as a socially learned practice through which ecological knowledge, technical expertise, and cultural traditions are reproduced across generations.

### 4.5. Values, Reciprocity, and Meat Sharing

The social organization of hunting is sustained by moral values that regulate cooperation, reciprocity, and the distribution of wild meat within Indigenous communities. Although hunting expeditions require individual abilities, successful outcomes depend on collective participation, mutual trust, and shared responsibilities among hunters and their families. These values are not external to hunting activities but constitute the ethical framework through which access to wildlife is socially regulated and community relationships are reinforced [39].

One of the clearest expressions of these principles is the customary sharing of *N. narica* meat after a successful hunt. Rather than being interpreted solely as food distribution, meat sharing represents a culturally embedded practice that strengthens kinship ties, reciprocity networks, and social cohesion. Hunters frequently distribute portions among relatives, neighbors, or participants in the hunting expedition, thereby extending the benefits of hunting beyond the individual who harvested the animal. Similar practices have been documented among numerous Indigenous and rural societies, where the circulation of wild meat functions as a mechanism for reinforcing social solidarity and reducing inequalities in access to valuable animal protein [40].

Reciprocity also influences harvesting decisions. The preference for taking only the number of animals required for household consumption or for collective sharing reflects an ethic of moderation that limits unnecessary extraction while recognizing wildlife as a shared component of community life rather than an unlimited commodity. Such practices do not necessarily emerge from formal conservation policies; instead, they arise from locally transmitted moral principles that integrate subsistence needs with long-term relationships between people, wildlife, and territory [41].

From a biocultural perspective, the distribution of hunted meat illustrates how food systems simultaneously reproduce ecological knowledge, social institutions, and cultural identity. Wild meat therefore possesses nutritional, economic, and symbolic value. Beyond its contribution to household diets, its circulation reinforces collective memory, intergenerational learning, and the reciprocal obligations that sustain community life. These findings coincide with broader ethnoecological research demonstrating that food sharing constitutes a fundamental mechanism through which Indigenous societies maintain social resilience while strengthening sustainable relationships with local ecosystems [25,27,42].

Thus, hunted meat should not be understood exclusively as a source of animal protein but as a biocultural food that materializes social reciprocity, ecological knowledge, and the historical relationships through which Indigenous communities inhabit and give meaning to their territories.

### 4.6. Human–Animal Cooperation in Hunting

The hunting of *N. narica* depends not only on hunters’ ecological knowledge and technical skills but also on long-established relationships of cooperation with domesticated animals. Dogs and horses are active participants in hunting expeditions, each performing complementary roles that enhance hunters’ capacity to navigate complex forest environments, locate prey, and transport game. Rather than functioning merely as auxiliary tools, these animals contribute to an integrated hunting system in which human and non-human actors cooperate through practices developed over generations [43].

Hunting dogs occupy a particularly important role within this system. Hunters recognize them for their ability to detect scent trails, pursue coatis through dense vegetation, and indicate the location of prey by persistent barking. Their effectiveness is not considered an innate characteristic alone, but the result of continuous training, accumulated experience, and close relationships established with their owners. Similar forms of human–dog cooperation have been documented in Indigenous hunting societies throughout Mesoamerica and other regions of the world, where dogs significantly increase hunting efficiency while simultaneously participating in processes of knowledge transmission and cultural continuity [44]. The incorporation of recent evidence highlighting the complementary roles of dogs in Indigenous hunting systems further supports the importance of these long-standing human–animal partnerships [45].

Horses perform equally significant, although different, functions. They facilitate access to remote hunting areas, reduce the physical demands of long-distance travel, and transport harvested animals across rugged terrain. Their contribution extends the spatial range of hunting activities and allows hunters to exploit forest landscapes that would otherwise be difficult to reach on foot. In this sense, horses should not be understood solely as means of transportation but as essential collaborators that shape the organization and logistics of hunting expeditions [46,47].

Taken together, these relationships illustrate how Indigenous hunting systems emerge from cooperative interactions among humans, domesticated animals, and forest ecosystems. Hunting success therefore reflects the integration of ecological knowledge, technical expertise, and interspecific collaboration rather than the actions of hunters alone. From a biocultural perspective, these long-term partnerships exemplify how cultural practices are co-produced through sustained interactions between people, animals, and their environments, reinforcing both hunting traditions and community resilience [5,25].

### 4.7. Community-Based Conservation

The findings indicate that the continued use of *N. narica* within Indigenous communities is accompanied by locally developed practices that contribute to wildlife stewardship. Although none of the interviewed hunters explicitly described their actions using the language of biodiversity conservation, their harvesting decisions are guided by culturally transmitted principles that regulate extraction, discourage unnecessary killing, and recognize the long-term importance of maintaining healthy wildlife populations. These practices illustrate how conservation may emerge through customary institutions embedded in everyday livelihood activities rather than through externally imposed regulations [48].

This form of community-based stewardship reflects a broader pattern documented in Indigenous territories throughout Mesoamerica, where traditional ecological knowledge and local governance contribute to the sustainable management of forests and wildlife [5,6,7,8]. In the communities studied, hunting is integrated into diversified livelihood strategies that include agriculture, forest use, and reciprocal social relations, reducing dependence on intensive wildlife extraction while maintaining the ecological functions of local ecosystems.

These observations support growing evidence that Indigenous knowledge systems can make significant contributions to biodiversity conservation by integrating ecological understanding with ethical norms and collective decision-making [49]. Recognizing such locally grounded management systems is therefore essential for developing conservation policies that strengthen, rather than replace, community governance of wildlife resources.

### 4.8. Wild Meat Culinary Heritage

Beyond its ecological and economic importance, *N. narica* occupies a meaningful place within the culinary traditions of northeastern Oaxaca. Wild meat is valued not only because it provides animal protein but also because it embodies culinary knowledge, family memories, and cultural identities that have been transmitted across generations. Hunting therefore culminates in a food system where ecological knowledge, food preparation, and social relationships converge.

The preparation and consumption of coati meat represent much more than the final stage of a hunting expedition. Culinary practices transform wildlife into biocultural foods whose value derives simultaneously from their nutritional properties, cultural significance, and the collective experiences associated with obtaining, preparing, and sharing them. Similar relationships between wild meat, food heritage, and cultural identity have been documented among Indigenous and rural communities throughout Latin America, where traditional cuisines continue to express long-standing interactions between people, biodiversity, and territory [50,51].

At the same time, recognizing the cultural importance of wild meat should not obscure the need to consider public health. The consumption of wildlife may involve exposure to zoonotic pathogens when animals are handled or prepared without appropriate sanitary precautions. Although this issue was beyond the scope of the present study, future interdisciplinary research integrating ethnoecology, veterinary sciences, and public health would contribute to a more comprehensive understanding of sustainable and safe wildlife use [52,53].

Ultimately, the culinary use of *N. narica* demonstrates that food systems are inseparable from the ecological and cultural processes that sustain them. Wild meat should therefore be understood not simply as a subsistence resource but as a biocultural food through which ecological knowledge, social reciprocity, historical memory, and territorial identity are continually reproduced.

Taken together, the findings demonstrate that the hunting of *N. narica* in northeastern Oaxaca constitutes a biocultural system in which worldview, ecological knowledge, technical expertise, moral values, cooperation with domesticated animals, community governance, and culinary traditions interact to shape human–wildlife relationships. Rather than representing independent dimensions, these components form an integrated socioecological network that has enabled Indigenous communities to adapt to changing environmental and economic conditions while maintaining culturally meaningful relationships with local biodiversity. By documenting these interactions, this study contributes to a broader understanding of how biocultural approaches can enrich wildlife research, strengthen community-based conservation, and highlight the role of traditional food systems in sustaining both biological and cultural diversity.

## 5. Conclusions

The hunting of *N. narica* in the four Indigenous communities studied encompasses a flexible combination of practices that include (a) diurnal collective hunting supported by dogs and horses, (b) crop protection through hunting and the use of animal-based repellents, and (c) limited and context-dependent commercialization of meat. These practices coexist and are selectively activated according to subsistence needs, agricultural cycles, and local socio-economic conditions. Likewise, the preparation of *N. narica* meat—through roasting, chile-based barbecue, broth, frying, and yellow mole—reflects the integration of this species into diverse traditional food systems rooted in regional culinary knowledge.

From a biocultural ethnoecological perspective, the documented uses of *N. narica* reveal a multifaceted human–animal relationship in which biological attributes of the species, such as body size, behavior, and habitat use, intersect with culturally specific knowledge, preferences, and values. *N. narica* is simultaneously perceived as game, food, crop pest, and occasional source of income, illustrating how local classifications and management strategies transcend rigid dichotomies between wildlife conservation and resource use.

More broadly, this study demonstrates that the hunting of *N. narica* in northeastern Oaxaca should not be interpreted merely as the extraction of a wildlife resource but as a biocultural system in which traditional ecological knowledge, subsistence practices, food systems, cooperation with domesticated animals, and community governance are dynamically integrated. By documenting these interactions, the study contributes to a broader understanding of how Indigenous hunting systems sustain both biological and cultural diversity while simultaneously supporting local livelihoods and food security. Beyond its regional relevance, this research highlights the value of biocultural ethnoecology as an analytical framework for understanding human–wildlife relationships and for informing culturally appropriate approaches to wildlife management and community-based conservation in Mesoamerica and other regions where Indigenous knowledge continues to shape biodiversity stewardship.

## Figures and Tables

**Table 1:** Hunting and meat use of *N. narica* in Indigenous locations in Northern Oaxaca, México.

Location (Indigenous Group)	Management	Meat Use	Recipe
Chimalapa (Zoque)	Diurnal hunting	Fresh	Roasted
Camotlán (Zapotec)	Diurnal hunting, crop protection	Fresh	Chile-based barbecue, broth
		Dry	Yellow mole
Ayotzintepec (Chinantec)	Diurnal hunting, crop protection, sale	Fresh	Broth, fried, roasted
Chilchotla (Mazatec)	Diurnal hunting, Crop Protection, Sale	Fresh	Chile-based barbecue, yellow mole, roasted

## Data Availability

The original contributions presented in this study are included in the article. Further inquiries can be directed to the corresponding authors.
